# The Food Microplastic Pyramid (FOMIC-Py) as a Novel Framework for Prioritizing Dietary Exposure and Industrial Processing Impact: An Italian North-South Exposure Model

**DOI:** 10.3390/toxics14070578

**Published:** 2026-06-30

**Authors:** Umberto Cornelli, Martino Recchia, Claudio Casella

**Affiliations:** 1 Department of Molecular Pharmacology and Therapeutics, School of Medicine, Loyola University, 2160 1st Ave, Maywood, IL 60660, USA; 2 Mario Negri Institute, Via Mario Negri 2, 20156 Milan, Italy; statmed@hotmail.com; 3 Department of Chemistry, University of Pavia, Viale Taramelli 12, 27100 Pavia, Italy; gz@unipv.it

**Keywords:** microplastics, food chain, food contamination, human health

## Abstract

Dietary exposure to microplastics (MPs) has emerged as a significant concern; therefore, its implications for exposure characterization are presented in this study. The lack of standardized testing methods currently limits effective risk management. Determining how industrial operations contribute to the presence of these xenobiotics in the food supply chain is essential, even if environmental absorption is a recognized factor. The Food Microplastics Pyramid (FOMIC-Py), a novel hierarchical structure designed to correlate MP prevalence with industrial processing intensity, is presented in this study. The investigation suggests that technogenic inputs may represent important contributors to contamination by synthesising current literature and applying the model to regional food patterns, especially an Italian North-South scenario study. The method uses sensitivity analysis (Spearman’s ρ = 0.94) for statistical validation and classifies food items from primary commodities (Level 1) to ultra-processed items (Level 5). Mechanical abrasion and packaging interactions are recognized as the main vectors by the FOMIC-Py, which reveals a consistent accumulation of MPs across all five levels of industrial transformation. While FOMIC-Py reliably assesses particles over 1 µm, current analytical constraints regarding nanoplastics lead to a significant exposure underestimation. Consequently, rather than being an established predictive model of human target-organ dosage, the FOMIC-Py framework serves as a new exploratory, hypothesis-generating tool. The absolute exposure metrics should be evaluated cautiously owing to the underlying variability of worldwide MP extraction data, even if our statistical predictions indicate a consistent relative ranking hierarchy across contaminated food categories. These first screening criteria provide a uniform basis to direct future targeted sampling procedures and regulatory prioritization.

## 1. Introduction

Global plastic production has reached unprecedented levels, with an estimated 430.9 million tonnes (Mt) produced in 2024, raising significant concerns for environmental contamination and public health [1]. All environmental matrices, including soil, water, air, and the world’s food chain, have now been found to contain microplastics (MPs). MPs are either directly introduced through industrial uses or result from the decomposition of these polymers [2,3]. Human exposure by ingestion, inhalation, and skin contact is facilitated by their small size, chemical diversity, and potential to absorb exogenous contaminants [4,5]. MPs were identified in several kinds of commonly used food items, including seafood, salt, sugar, dairy, cereals, and beverages, thus enabling a more precise characterization of dietary exposure patterns across different populations [6,7,8,9].

Food processing and food contact materials are becoming increasingly recognized as significant routes for MP contamination outside of environmental sources. During primary manufacturing, storage, transportation, and preparation, plastic packaging materials and processing equipment may introduce particles into food [9,10,11]. MP loads in food structures are modulated by processing conditions and packaging type, as demonstrated by migration from food contact surfaces, containers, and thermal or mechanical treatments in a number of products, from packaged sugars and salts to bottled beverages and prepared meals [12,13,14,15].

MP contributions from processing and contact materials may be systematically quantified according to the FOMIC-Py analytical framework. This study extends past descriptive monitoring by setting forth a mechanistic framework. FOMIC-Py suggests that connections between food processing and packing might be significant factors influencing dietary MP exposure. By integrating empirical migration data, polymer-specific signatures, and processing-dependent release patterns, the FOMIC-Py framework enables the systematic attribution of MP loads to non-environmental sources, supporting both exposure assessment and regulatory prioritization.

According to mechanistic studies, MPs may function as carriers for additives, persistent organic pollutants, and microbial biofilms in addition to promoting inflammation, oxidative stress, endocrine disruption, and immunological modulation. These pathways are consistent with new epidemiological and medical findings. The cardiovascular, metabolic, pulmonary, gastrointestinal, reproductive, and neuroinflammatory systems are among the chronic and acute disease categories that may require further study pertaining to MP exposure, according to previous exploratory studies. The Chicago Cluster framework, which was used to structure these findings, should be considered a conceptual model that generates hypotheses rather than providing definitive proof of causal or clinically verified relationships [6]. The combined effect of mechanistic and population-level signals highlights the need for quantitative exposure evaluations even while causal connections are still being investigated.

Dietary intake constitutes a primary route of MP exposure, yet regional variation in consumption patterns, food processing intensity, and contamination levels remains poorly characterized [5,8,16]. Italy provides a particularly relevant model, given its pronounced North–South heterogeneity in dietary habits, food sourcing, and food system characteristics. Evaluating population-level exposure, identifying crucial supply chain control points, and developing specific mitigation strategies all depend on an understanding of these disparities.

This study proposes a comprehensive integrative review that combines estimates of MP concentrations across 56 food categories with harmonized food consumption data. By linking region-specific dietary patterns with standardized particle counts, we provide a data-driven assessment of annual MP intake in Northern and Southern Italy. In order to improve dietary exposure and future food system modifications, the present study aims to (i) quantify regional differences in dietary MP exposure, (ii) identify dominant food contributors, and (iii) establish a replicable methodology for integrating various data sets. This method provides an original perspective on the impact of food processing and supply-chain pathways on MP contamination, as well as differences in exposure.

The Scopus, Google Scholar, PubMed, and ISI Web of Science databases were searched to compile the bibliographic selection of publications published until April 2026.

The literature search was conducted according to PRISMA principles. The following Boolean strategy was adapted for each database: microplastic OR plastic particle AND food OR beverage OR diet AND exposure OR contamination OR intake. Two investigators independently screened titles, abstracts, and full texts. Disagreements were resolved by consensus. Only peer-reviewed English-language studies reporting quantitative concentrations in edible food matrices were included. A total of 55 studies fulfilled eligibility criteria and were incorporated into the harmonization process [17,18]. There are not 56 distinct studies that correspond to the 56 food categories that were reviewed. While certain categories required harmonization across multiple sources or exploratory proxy-based modeling where empirical data were lacking, others were informed by several publications. Studies that reported only environmental samples without edible-food measurements, lacked polymer confirmation by spectroscopic or comparable analytical techniques; reported only qualitative findings; or were conference abstracts, editorials, or reviews without primary quantitative data were all excluded. All pertinent studies that were indexed up until April 2026 were incorporated in the search process.

All authors went over and examined the accepted articles. Additionally, the collected publications’ reference lists were reviewed to find additional relevant studies. Detailed information on search strategies, inclusion and exclusion criteria, and the articles included in this review are summarized in Figure S1, which depicts the PRISMA flow diagram.

## 2. Annual Household Food Consumption and Harmonization of MP Concentration

The Computer-Assisted Personal Interviewing (CAPI) dataset [19], a nationally representative survey of 19,500 families throughout Italy, was used to calculate the annual household food consumption (kg per household). To normalize consumption estimates and guarantee cross-regional comparability, the sample’s average household size was 2.3 persons. To estimate the yearly quantity of MPs per household in Northern and Southern Italy, annual quantities for each of the 56 food groups were taken as weighted averages from the CAPI dataset and then combined with MP concentrations derived from literature. The Italian Society of Human Nutrition (SINU) was used to establish the serving sizes [20,21,22,23]. For solid foods, all values were converted to MPs/kg, and for beverages, MPs/L. No assumptions were made regarding density; conversions were strictly unit-based (MPs/g → MPs/kg). Data were converted to MP/kg only in instances where the edible mass was explicitly stated in publications reporting concentrations per item (i.e., fruit).

A number of recurring numerical trends were observed in the particle count values provided for each of the 56 food categories, with certain categories sharing nearly identical estimates. The utilization of median or mean values from several worldwide studies, many of which report concentrations of comparable order of magnitude despite methodological fluctuations, shows up in this convergence. Individual estimates could differ by more than an orders of magnitude around the main trend due to the underlying datasets originating from different countries and analytical methods.

### 2.1. Derivation and Assumptions of Modeled Measures (MM)

For every food category, descriptive statistics were determined and presented as means and standard deviations. Standard deviations represent the variability derived from literature-reported concentration ranges and consumption datasets.

A standardized Process-Packaging Proxy technique was employed to derive Modelled Measures (MM), which serve as proxy-based exploratory estimates for food categories that had no empirical data in peer-reviewed literature. We established reference clusters of foods with the same moisture profiles, mechanical shear inputs, environmental matrices, and packaging materials. For instance, the ‘cooked vegetable-and-pasta’ cluster (empirical range: 175–1350 MPs/kg) was mapped onto pre-packaged soups [MM: 1450 MPs/L]. In order to account for the combined technogenic inputs of high-shear mixing, thermal retorting, and extended contact with plastic-lined multilayer pouches, the modeled mean was purposefully placed in the highest quartile of the cluster range. Unit conversions assumed conservative limitations to avoid exposure underestimation by rigorously adhering to mass-unit restrictions without arbitrary density scaling (calculation explained in Table S1) [18]. A category was assigned MM when no peer-reviewed study quantified MPs in the edible serving of that food, available data referred only to packaging, environmental matrices, or non-edible tissues, or values could not be inferred without introducing unvalidated assumptions (MMs are hypothetical estimations based on heuristics) [11]. When direct empirical data were not available, MM values were obtained by applying a structured process-packaging proxy technique. Reference food clusters with comparable environmental sources, processing magnitudes, packaging interactions, and matrix properties have been employed in this approach. Therefore, rather than being direct measurements, MM values should be considered to be exploratory exposure estimates. This approach ensured that the exposure model was based on reliable literature-based data concentrations while establishing an explicit distinction between categories for which there remained an overall lack of proof as well as values that were confirmed by empirical data.

Table S2 presents representative central values (mean or median, depending on availability) for the purpose of harmonization, along with reference numbers that identify the studies that support every category of food. Strong regional exposure assessments have been rendered feasible by these harmonized data, providing a comparable and uniform baseline rather than an in-depth representation of global variability. Table S3 illustrates the numerical distribution of the subcategories for each food category and offers an in-depth overview of the diversity of food products across all of the commodity categories. The regional exposure divergence is strictly grounded in the different dietary patterns reported in national food consumption databases (Table S3), rather than geographic environmental variance alone. Table S4 presents discriminant analysis results for main food categories according to geographical area (Northern Italy vs. Southern Italy).

### 2.2. Statistical Analysis

For every food category, descriptive statistics were determined and presented as means and standard deviations. Standard deviations represent variability derived from literature-reported concentration ranges and consumption data and do not correspond to biological inter-individual variability.

The Shapiro–Wilk test was used to determine whether data distributions were normal prior to inferential analyses. Non-parametric descriptive summaries were mostly employed to describe central tendency and dispersion since MP concentration data indicated significant deviations from normality (*p* < 0.05). To minimize dimensionality and facilitate multivariate comparison across geographical regions, food items were combined into nine major dietary groups.

Statistical analyses were split into two distinct steps to ensure mathematical rigor. First, exploratory Linear Discriminant Analysis (LDA) was performed on the aggregated consumption-weighted household database to identify major macro-regional patterns between Northern and Southern Italy; due to the aggregated nature of this baseline matrix, formal cross-validation was omitted for this step. Second, to address non-normality and validate the regional pattern separation, a non-parametric Random Forest (RF) classifier and a Partial Least Squares Discriminant Analysis (PLS-DA) were implemented. For these machine learning workflows, a stratified 10-fold cross-validation architecture was strictly applied, utilizing the expanded scenario matrix (incorporating literature concentration ranges) to assess classification accuracy, sensitivity, and Cohen’s Kappa without overfitting. Rather than predictive classification, the aim was dimensionality reduction and pattern identification [24,25,26]. To guarantee generalisability and avoid overfitting on the combined dataset, the RF model was evaluated using a stratified 10-fold cross-validation approach. To supplement the LDA structural matrix loadings, Out-of-Bag (OOB) error rates and Variable Importance Measures (VIM) were obtained [24,25,26].

The 56 food items were pooled into nine categories, and then separate linear discriminant analyses were carried out (Table 1). A sensitivity analysis was carried out by comparing minimum and maximum literature values for each of the 56 categories in order to account for the inherent heterogeneity in MP reporting. The ranking of food items exhibited a Spearman correlation of *ρ* = 0.94 (*p* < 0.001), indicating the durability of the FOMIC-Py hierarchical structure, even while absolute concentrations fluctuated significantly (Wilcoxon test, *p* < 0.001) (Table S5). Table salt was excluded from the multivariate statistical models (LDA, PLS-DA, and Random Forest) despite the fact that the full dietary matrix includes 56 food groups, considering its extraordinarily high baseline content would significantly bias the categorization. In order to guarantee mathematical rigor and avoid artifactual classification, the current statistical dataset employs the use of a standard 55 operational variables.

SAS software (version 9.4; SAS Institute Inc., Cary, NC, USA) was utilized for every statistical analysis. Statistical significance was determined at α = 0.05, and two-sided tests were performed. To facilitate straightforward understanding of geographical differences, findings are provided with corresponding *p*-values where suitable.

### 2.3. Data Quality Scoring and Harmonization

Each data point was cross-validated using the quality criteria outlined in the Supplementary Materials [7,27,28,29,30,31,32] (Tables S6–S9) to minimize the inherent variability of analytical methods (i.e., μFTIR spectroscopy, or µFTIR; and μRaman spectroscopy, or µRaman). Only studies that included quantitative concentration data, sample preparation techniques, polymer confirmation, and specified contamination-control protocols were considered acceptable for harmonization. During the screening phase, studies that did not satisfy these minimal standards for analytical quality were eliminated. This screening protocol guaranteed that only studies with rigorous polymer identification and contamination control were included. To prevent skewness from extreme outliers in the literature, a Winsorization approach was applied to the upper 5% of reported concentrations before calculating the harmonized means for each category.

## 3. Results

Across the 56 food categories analyzed, literature-derived concentrations of MPs spanned several orders of magnitude (Table 2). Experimental data for several food categories were either unavailable or insufficient to enable direct quantification. In these instances, indicative estimations based on reported concentration data from similar food matrices and standardized serving sizes were obtained using MM. Instead, they are utilized to demonstrate possible exposure patterns under standardized consumption assumptions and to support relative comparisons among food categories.

Due to variations in food processing, packaging, and analytical techniques, solid food matrices indicated the most variability, with reported quantities ranging from negligible levels to substantial particle loads. Compared to solid food categories, MP concentration distributions in beverages and liquid matrices exhibited fewer differences. The harmonized median or mean particle counts presented in Table 2 are obtained from a selection of worldwide studies. Depending on the nation of origin and analytical method, reported variability usually ranges from 20 to 40%. Recurring or identical values indicate convergence among readily available information rather than data duplication. For Northern and Southern Italy, the mean with SD yearly consumption of MPs per household across all 56 food groups was determined using these harmonized MP concentration values (Table 3).

Different regional exposure patterns were identified: households in the North consumed more of several kinds of high-consumption processed and dairy products, while households in the South ingested less of certain basic foods, indicating sourcing and consumption patterns unique to the region. The findings suggest that, in comparison to the profile in the South, families in Northern Italy have a higher annual dietary exposure to MPs (approximate 9% higher), mainly due to the ingestion of dairy and processed foods more frequently. While most categories exhibit overlapping distributions, confirming a common baseline of contamination throughout Italy, these data indicate that dietary habits and food chain characteristics largely explain regional differences in MP exposure. The greatest MP concentrations were identified in potatoes (310) and crustaceans (550), suggesting that soil-to-root transfer, post-harvest washing procedures, and marine bioaccumulation constitute key pathways for plastic ingestion. The first seven foods in the ‘High’ category (≥100 MPs/serving) represent approximately 50% of the total MP intake that has been reported. This suggests that human exposure levels could be greatly decreased by dietary changes or more stringent packaging laws that target those specific matrices (Table 3).

The results indicate that the level of industrial processing and MP concentration were strongly correlated. Particle concentrations were considerably higher in ultra-processed or pre-packaged foods like canned meat (240 MPs/serving) and pre-packaged soup (218 MPs/serving) than in minimally processed foods like dry legumes (3 MPs/serving) or citrus fruit (4 MPs/serving). These preliminary observations are consistent with the hypothesis that the main sources of foodborne plastic contamination are mechanical processing and the migration of polymer-based packaging (Table 3).

### 3.1. Microplastic Concentrations and Construction of the FOMIC-Py

**Figure 1:**
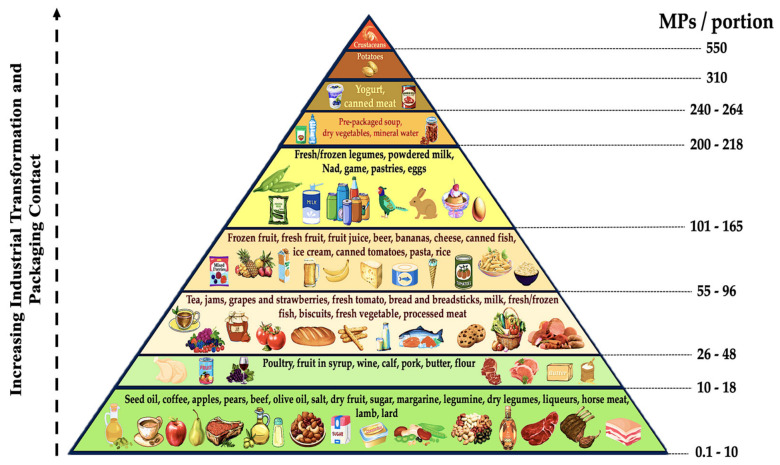
FOMIC-Py; dietary pyramid of food categories ranked by MP intake per serving. The FOMIC-Py framework. Levels are categorized by MP density, validated by a Spearman rank correlation (*ρ* = 0.94) between minimum and maximum literature scenarios. Food products have been organized into 5 hierarchical levels by the FOMIC-Py using a dual-metric approach: the degree of industrial processing/packaging interaction and the median MP concentration (MPs/kg or MPs/L).

The 56 food categories’ MP concentrations were determined using a combined empirical–modeled methodology. While data-poor classes were established by analogy with reference clusters sharing important exposure factors, published values were extracted and harmonized. To generate modeled ranges, cluster ranges were cautiously increased, and midpoints were utilized as MMs. The FOMIC-Py, an organized framework for displaying and analyzing MP exposure across food categories, is statistically based on this harmonized matrix (Figure 1). The reported data was compared to the model predictions for foods with known or published MP content. The Modeled Ranges’ reliability and uniformity with empirical data were thus confirmed. Due to variability in sampling, digesting procedures, polymer identification, particle size cut-offs, and reporting units, reported MP concentrations in the literature are discordant. The present study employed a straightforward, explicit modeling technique to make comparisons obvious and repeatable: the MP concentration (MP mean) for each food or beverage. Recognizing that these values are estimates of orders of magnitude rather than absolute measurements, the central value weighted by the analytical robustness of the selected studies was used.

Since there are currently no established biomarkers of human dietary MP intake, the FOMIC-Py cannot be directly verified versus biological biomarkers of exposure. Despite the fact that MPs have been found in human tissues and feces, the information currently available does not provide attribution to specific food sources. Consequently, indirect validation was carried out by evaluating the coherence of the model results with known contamination pathways, such as increased MP concentrations in packaged beverages, ultra-processed meals, and foods that have been in contact with plastic materials for long periods of time. Furthermore, contamination processes associated with packaging and processing are consistent with the prevalence of polymers like polyethylene (PE), polypropylene (PP), and polyethylene terephthalate (PET) across food categories. FOMIC-Py represents a robust risk management and prioritization tool. Beyond its conceptual value, it provides a functional framework for food safety authorities to identify high-risk matrices and implement targeted mitigation strategies within the food supply chain.

Similarly with the underlying studies, it expresses data in MPs/kg for solid/semi-solid foods and MPs/L for beverages. Values were determined by comparison with closely comparable food categories in situations where direct min–max data were not available; these instances are specifically noted to preserve traceability and transparency. The central pattern of reported data was represented by an operating range for every food category. These ranges represent representative values derived from multiple independent studies. Severe outliers associated with atypical production chains or individual brands were excluded. To prevent underestimating analytical variability, thereby artificially shortening the intervals. Consequently, operational ranges are designed to simplify exposure modeling while maintaining scientific transparency; therefore, they’re representative rather than exhaustive. Using harmonized concentration data and serving sizes established for the Italian population, food groups are arranged within the pyramid based on increasing MP burden per serving. The relative contribution of frequently consumed foods to dietary MP exposure is demonstrated by this visual scheme and provides a straightforward means of comparing food categories.

### 3.2. The Hierarchical Distribution of MPs

The cumulative “surface-contact time” throughout industrial cycles (such as homogenization, high-speed bottling, and heat-sealing of polymers) coincides with the increasing trend in MP concentration observed in the FOMIC-Py levels, indicating these as crucial control points for mitigation (Figure 1).

MP prevalence appears to increase with food supply chain complexity, according to the FOMIC-Py’s hierarchical structure (Figure 1, Table 3). The cumulative “plastic footprint” extends considerably when food passes from Level 1 (Raw/Minimal) to Level 5 (Ultra-processed).

**Figure 2:**
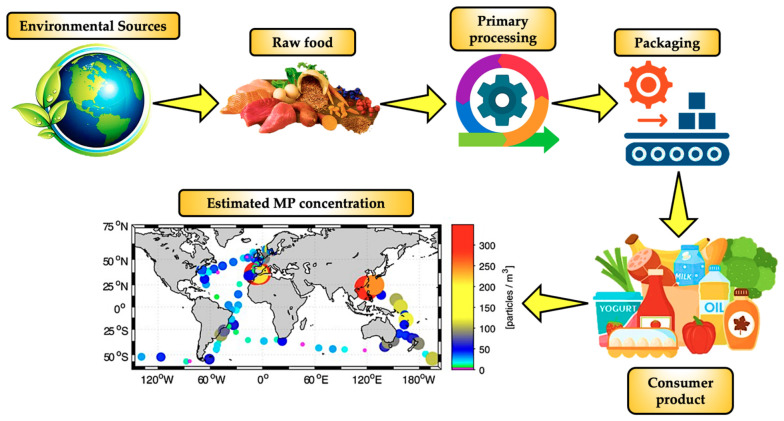
Conceptual representation of the principal contamination pathways considered within the FOMIC-Py.

Figure 2 summarizes the main contamination pathways included in the FOMIC-Py model.

This pattern suggests that MP contamination is mainly caused by technogenic inputs during industrial phases, such as mechanical abrasion of bottling equipment, atmospheric fallout in processing plants, and the leaching of micro-polymers from synthetic packaging under mechanical or thermal stress, rather than just environmental uptake (i.e., in crops or livestock). Raw foods are nonetheless susceptible to the presence of MPs despite their not having undergone industrial transformation due to ubiquitous environmental stressors like polluted aquatic habitats, plastic-laden fertilizers, and soil deterioration.

The implementation of the FOMIC-Py framework exhibits a clear trend of intensification whereby the median MP concentration increases in direct proportion to the complexity of the food supply chain, revealing a distinct stratification of MP occurrences across the 5 established levels. MP concentrations were generally lowest in Levels 1 and 2 (Baseline and Low Processing), with Level 1 (Raw/Untreated) commodities acting as an environmental baseline driven primarily by external uptake rather than industrial intervention. However, a significant rise in MP presence was observed in the intermediate categories. Most notably, Level 4 commodities indicated a substantial rise, indicating that industrial handling and secondary packing constitute significant polymer migration vectors. Overall, the Level 5 (Ultra-Processed) group achieved the greatest exposure levels, indicating potentially important contributors to dietary MP exposure.

A Wilcoxon signed-rank test for related samples was carried out to compare the low exposure scenario (minimum reported values) to the high exposure scenario (highest reported values) for the 56 categories in order to demonstrate that the model is resilient to variability in literature data. The findings of the sensitivity analysis demonstrated that the minimum and maximum exposure scenarios had a Spearman rank correlation of ρ = 0.94 (*p* < 0.001). It is crucial to emphasize that the FOMIC-Py model findings are not biologically or toxicologically validated by this strong correlation. Rather, it provides a mathematical demonstration of the stability and structural integrity of the hierarchical ranking criteria, demonstrating that even with the large numerical fluctuations in raw literature data, the relative classification of food items from lowest to greatest plastic burden remains constant (Table S5).

Italy’s reported national food consumption patterns are consistent with the regional dietary exposure patterns to MPs found in this study. Recent data from the National Food Consumption Survey IV SCAI suggests that, considering substantial regional differences in consumption patterns throughout the nation, cereals and cereal-based products still continue to be the main sources of energy intake, followed by milk and dairy, oils and fats, and meat products [19,110].

Significant variations between Northern and Southern Italy were found employing linear discriminant analysis when the 56 food items were divided into nine pooled categories (Table 4). Salt was not included in the analysis with the other food items because it is also used in the preparation of certain dishes (such as salt-crusted chicken or sea bass) and in cheese production. For this reason, its value would have been overestimated.

For the majority of food categories, significant canonical discriminant functions were identified, indicating distinct regional consumption patterns. Vegetables and legumes, dairy products, drinks, and meat and animal derivatives exhibited the strongest regional discrimination, as seen by their low Wilks’ lambda values and high canonical correlations. Conversely, fats, seasonings, and cereal-derived products showed a weaker discriminatory effect. The most discriminating categories were dairy and vegetables, which contain from 35 to 264 MPs/serving.

The polymeric composition of MPs varied across food categories but followed consistent patterns associated with packaging and processing materials. As summarized in Table 5, the most commonly detected polymers were identified in food categories exhibiting the highest concentrations of MPs.

**Figure 3:**
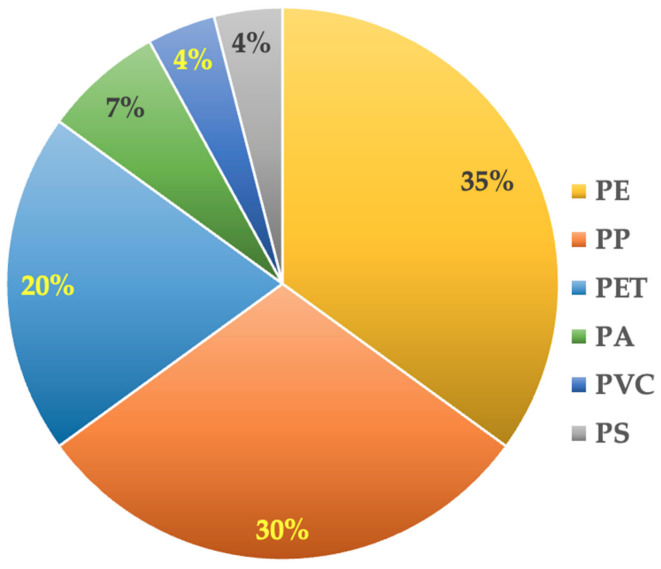
Predominant polymer composition of MPs in representative food categories. Percentages cannot be considered to serve as quantitative meta-analytic estimations; rather, they are simply schematic representations generated from qualitative frequency patterns observed throughout the examined studies [41,55].

The predominant polymer composition across representative food categories is shown in Figure 3.

In summary, materials frequently used in food processing and packaging clearly predominated in polymeric profiles across food categories. The predominant polymers found in fruit and vegetables, non-alcoholic drinks (Nad), dairy products (milk, cheese, and yogurt), and mineral water were consistently found to be PE, PP, and PET. Throughout the food supply chain, this recurrent pattern suggests a common origin associated with handling practices, packaging materials, and contact with industrial surfaces. In contrast, fish products exhibited a more heterogeneous polymeric composition, including PE, PP, polyamide (PA), polyvinyl chloride (PVC), and polystyrene (PS).

At the household dimension, the annual MP intake was established using an average family size of 2.3 members. Table 6 provides, in descending order, the estimated annual MP intake per family for each of the 56 categories of food that have been examined.

Table 6 indicates that there were statistically significant differences in annual MP intake between Northern and Southern Italy for several food categories; no discernible regional pattern was identified. Significant differences have been identified when twenty-four food classes were analyzed regionally; seventeen classes exhibited greater levels in the North and seven in the South. The distributions exhibited significant overlap across multiple orders of magnitude, despite the fact that overall intake was around 9% higher in northern regions. These findings add to the growing body of literature demonstrating that dietary exposure to MPs varies substantially among populations, as further detailed in Table 6.

### 3.3. Multivariate Analysis of Regional Dietary MP Exposure

MP concentrations fluctuated by more than two orders of magnitude among dietary groups, according to descriptive analysis. The median MP levels of minimally processed matrices were lower compared to that of prepared meals and drinks. Cereals and cereal-based items, dairy products, drinks, and processed meat derivatives contributed the most to the projected total MP intake when grouped into nine significant dietary categories.

**Figure 4:**
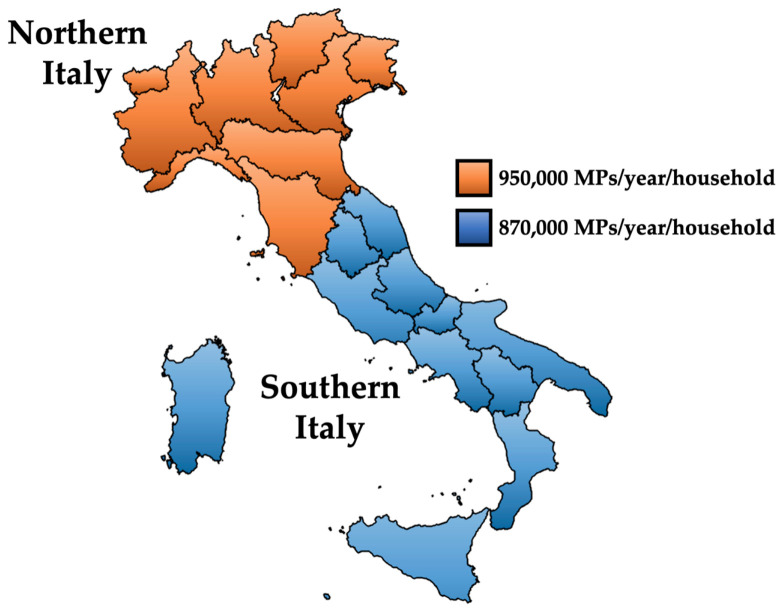
Northern and Southern Italy’s estimated yearly dietary MP exposure is spread regionally. By combining household food consumption information from the Italian CAPI survey with harmonized MP concentrations from the literature, regional estimates were generated.

Northern and Southern Italy exhibited a substantial multivariate separation (Wilks’ λ = 0.021, *p* < 0.001) according to LDA. 94.7% of the variance across groups was explained by the initial discriminant function. Cereals and cereal-based foods were more closely related to the Southern nutritional profile, while vegetables and legumes, dairy products, beverages, and meat derivatives emerged as the principal contributors to regional differentiation. The discriminant function and regional grouping were strongly associated, as evidenced by the high canonical correlation (r = 0.989). Figure 4 exhibits the estimated dietary MP exposure’s regional distribution. Figure 4 exhibits the estimated dietary MP exposure’s regional distribution.

The yearly household MP exposure in Northern Italy is projected to be 9% higher than in Southern Italy, as illustrated in Figure 4. Findings should be regarded as an indication of relative food exposure patterns, as they represent exposure estimates rather than observed biomarker values.

The regional differentiation observed by LDA was validated by partial least squares discriminant analysis (PLS-DA). The first latent variable explained 87.3% of the variance in the response variable and efficiently distinguished the groups. An overall classification accuracy of 91.4% was obtained through cross-validation, with a reasonable model fit (R^2^Y = 0.89) and internal model consistency (Q^2^ = 0.82). Vegetables and legumes, dairy products, drinks, and meat derivatives were exhibited to be the major contributor to regional differentiation (VIP > 1.0) according to variable importance in projection (VIP) scores. Regional variations in MP intake patterns are primarily influenced by only a small percentage of dietary groups, as evidenced by the partial overlap of lower-impact food categories. LDA and PLS-DA findings should be considered exploratory pattern-recognition tools, while the underlying dataset was constructed using harmonized and partially modeled category-level values rather than individual observations. They don’t represent dietary exposure’s external predictive validity [18].

### 3.4. Non-Parametric Machine Learning

A non-parametric RF classifier was implemented over the 55 food variables using a stratified 10-fold cross-validation architecture in order to handle any distributional anomalies and the high variability of the dietary microplastic data matrix. With an overall cross-validated accuracy of 92.59% and a Cohen’s Kappa value of 0.8518, the RF model effectively supported the robust macro-regional exposure segmentation previously proposed by the LDA (Tables S6–S8). The most significant empirical factors influencing the regional variation in human MP ingestion profiles were “Vegetables,” “Legumes,” and “Dairy Products,” according to the VIM assessed using Mean Decrease in Gini (MDI) (Table S9).

## 4. Discussion

The significant connection between eating habits and potential exposure to contaminants is demonstrated by the fact that these fundamental dietary categories are also recognized as major contributors to MP intake [6,41]. A structural study of dietary exposure has replaced descriptive contamination-harmonized surveillance with the creation of FOMIC-Py. These results demonstrate that MP ingestion is in accordance with the hypothesis that the prevalence of dietary MP may be significantly influenced by industrial food processing. A cumulative MP concentration incurred during industrial cycles—specifically through high-speed bottling, heat-sealing, and extended contact with polymeric surfaces—is reflected in the pyramid hierarchy’s transition from primary products (Level 1; Figure 1) to ultra-processed dairy and complex proteins (Levels 4–5; Figure 1). Our sensitivity analysis (Table S5, *ρ* = 0.94) confirmed the FOMIC-Py’s stability, indicating that industrial processing appears to be associated with higher MP concentration across several food categories. Our holistic model suggests that processed dairy and industrial beverages may contribute a larger particle load to the modern diet, whereas earlier research concentrated on single-matrix contamination (such as seafood or salt). These findings are consistent with the “Process-Packaging Proxy” hypothesis, which proposes that mechanical processing and packaging-related interactions may contribute to the transfer of MPs into food matrices.

A paradigm transition from environmental to technogenic contamination promotes the concept that technogenic sources may be involved in environmental contamination pathways (Table 3). The crucial concentrations observed in Levels 4 and 5 correspond to a processing-packaging relationship, even though Level 1 commodities are naturally vulnerable to MP accumulation due to widespread environmental stressors, such as the translocation of particles from plastic-mulched agricultural soils or contaminated irrigation water. The breakdown of polymer-based packaging during heat-sealing and mechanical abrasion from industrial machinery emerge as crucial contamination pathways in these highest categories, indicating that the more an item is “transformed,” the more significant its cumulative plastic footprint.

The highest level regarding potential human exposure is represented by Level 5 of the FOMIC-Py framework, where industrial processing and sophisticated packaging technologies are more common contamination vectors than environmental bioaccumulation [48,111]. The unexpected thermo-mechanical breakdown of polymeric membranes during high-speed production cycles is the main mechanism of MP penetration in these ultra-processed cohorts. In particular, multi-layer plastic films—usually PE, PET, or polymer-aluminum laminates—are exposed to extreme thermal gradients during processes including heat-sealing, thermoforming, and aseptic filling. This process may induce localized polymer chain scission and affect the material’s crystallinity, thereby increasing its susceptibility to microscopic fragmentation [48,111]. Additionally, the extreme mechanical stress produced by high-pressure pumps and high-frequency vibrations on automated assembly lines generates considerable surface abrasion on the interior walls of containers during the preparation of drinks and ready-to-eat meals. MPs and nanoplastics (NPs) are dislodged directly into the food matrix by this “scouring” process, which is facilitated by kinetic friction and fluid turbulence [112]. Additionally, the study indicates that closure geometries including heat-pressed induction seals and high-density polyethylene (HDPE) screw caps act as critical point sources; the torque generated during mechanical capping and the ensuing friction upon opening produce large amounts of shear-induced debris [113]. In industrialized regions such as Northern Italy, where highly automated processing is a significant risk factor for global food safety, these technological factors clarify why MP density in Level 5 products exceeds baseline environmental concentrations [46,107].

**Figure 5:**
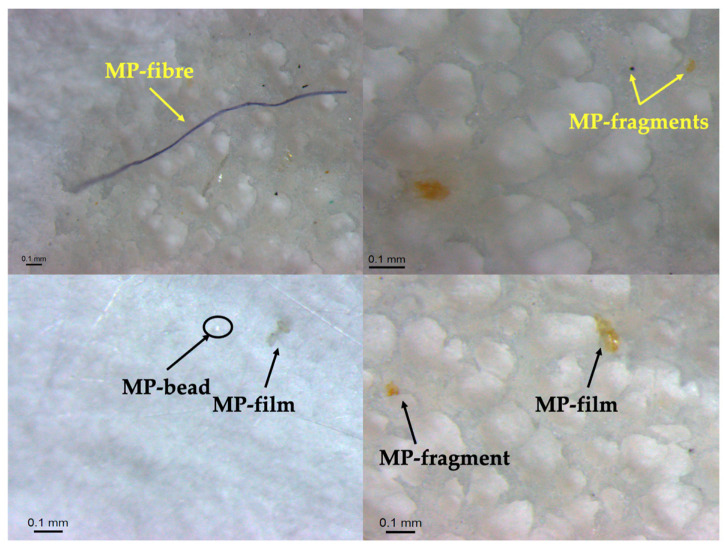
Common MP morphological classification observed in food matrices (fibers, fragments, films, and beads) under stereomicroscope [114].

Nevertheless, it is essential to understand that these hierarchical data represent a fairly conservative baseline; the reported concentrations, especially for high-intensity Level 5 products, probably only represent the underestimated percentage of total plastic ingestion due to the analytical detection limit of 1 µm and the exclusion of the NPs fraction. The analytical threshold for particle size detection is a crucial limitation of the existing exposure model. μFTIR or μRaman spectroscopies, which usually have a lower limit of detection (LOD) of about 1 µm, were used in the majority of investigations synthesised in the FOMIC-Py framework. Figure 5 illustrates representative MP morphologies that are frequently described in food matrices.

As a result, this review mainly excludes the possible influence of NPs. Additionally, the socio-regional implications are noteworthy due to the consumption of these ultra-processed commodities, which could be responsible for a significant amount of the MP intake difference among Northern and Southern Italian diets. This indicates that dietary patterns that lean toward higher-level items facilitate a significantly elevated risk of chronic MP exposure.

Due to their high reliance on grains, dairy, and plant foods, Italian dietary patterns are consistent with more general European Mediterranean models [23,115]. This suggests that widely consumed foods, rather than specialty or exotic foods, are the main exposure pathways for MPs. Previous studies demonstrating that MPs can be detected in fruit, vegetables, seafood, meat, dairy, and cereals, frequently at higher levels in processed food matrices [8,116,117], and systematic analyses reporting daily MP intake ranges from extremely low estimations in individual foods to very high values in beverages like bottled water confirm this perspective [5]. In this context, the application of FOMIC-Py provides an essential interpretative layer for understanding these exposure patterns.

Despite an overall tendency toward dietary standardization, the regional variations in pooled food category intake (Table 4) demonstrate that Northern and Southern Italy continue to have different nutritional patterns. Fruit, dairy products, vegetables and legumes, and beverages all significantly further regional disparities, indicating differences in nutritional supplies, dietary quality, and MP/NP content that might prove significant from a public health standpoint. These variations will probably occur from various intakes of fiber from food, bioactive compounds, animal-derived nutrients, and MPs, which are known to influence metabolic and inflammatory pathways, even if causal connections cannot be determined from the present study. Particularly interesting is the 9% greater yearly exposure observed in Northern Italy than in the South. This disparity is nutritional as well as geographical. A larger cumulative MP intake is the outcome of the Northern dietary profile, which is defined by a greater frequency of Level 4 and 5 foods (processed dairy and convenience products). These findings offer an exploratory context for future hypothesis testing. Neither the “Chicago Cluster” [6] nor the Microplastic Syndrome framework should be interpreted as a validated clinical construct. Both are presented exclusively as hypothesis-generating conceptual frameworks intended to guide future studies. According to this standpoint, the food category patterns observed in Table 4 may be associated with determinants of metabolomic phenotypes related to the risk of inflammatory and cardiometabolic diseases at the population level. It is important to interpret these results cautiously, nevertheless. The discriminant analysis was based on pooled food categories, which does not take serving size or total energy intake into consideration and may obscure within-group heterogeneity. Furthermore, inferences on temporal trends or health outcomes are not possible due to the cross-sectional nature of the data. To better understand the functional importance of these regional dietary variations and their possible influence on dietary exposure, further studies combining dietary evaluation with microbiota profiling and metabolomic characterization will be crucial.

It is crucial to make clear that the FOMIC-Py is not a clinical diagnostic tool but rather an exposure assessment tool. Nonetheless, the “Microplastic Syndrome” theory requires an empirical basis due to the high exposure levels seen in specific populations [6]. In this context, the long-term consumption of MPs—often more than thousands of units per year—may act as a chronic initiator for the metabolic and inflammatory changes hypothesized in our previous studies. FOMIC-Py enables targeted biomonitoring and the eventual validation of these clinical hypotheses by determining which food categories contribute more to this “MP load.”

From the perspective of public health, the identification of the food categories that most significantly differentiate regional dietary patterns (Table 5) could help develop focused nutritional policies meant to address unfavorable trends while maintaining advantageous conventional eating habits. When there is a reasonable belief compatible with the theoretical framework of microplastic syndrome, such as a “working hypothesis” [6], the finding that 7 food classes represent roughly fifty percent of the total MP intake may provide information about diet modifications; alternatively, products that may increase the MP excretion with feces may be administered [41]. These findings highlight two key public health implications. First, population-wide consumption patterns represent a significant part in estimating exposure to dietary MPs, which indicates that typical everyday foods may contribute a greater percentage of total intake than a single high-contamination item. Second, the quantitative data gaps for high-intake categories and the significant range in contamination levels suggest that the current exposure estimates are certainly prudent and underestimated real intake. In order to accurately estimate human exposure and evaluate potential health impacts, more reliable, harmonized data are required [118].

The additive effect of multi-matrix exposure is frequently ignored by current legislation. According to our approach, instead of concentrating only on reducing environmental sources, mitigation methods should give priority to the “Critical Control Points” of industrial activity. The dietary MP burden could be considerably reduced in Level 4–5 food production by reducing plastic-to-food contact time and replacing it with polymeric sealing.

Overall, the compositional patterns presented in Table 4 provide plausibility to the hypothesis that exposure to MPs in food is mainly caused by upstream industrial and packaging-related processes, whereas environmental sources are likely to be considerably more significant in aquatic foods. The observed regional variations emphasize the importance of integrating MP contamination into comprehensive assessments of environmental health and food safety, especially in regions that have significant consumption of foods that are prone to accumulating plastic particles. These exposure levels align with the exposure-prioritization categories defined in the Microplastic Syndrome framework [6], suggesting a need for targeted biomonitoring in these populations. Long-term exposure implications are much less common in Southern Italy [119]. Northern households consume 9% more MP annually, according to the Italian North-South exposure model. Different dietary patterns are statistically responsible for this divergence: the Southern diet maintains a larger frequency of unprocessed primary items, whereas the Northern diet is more dependent on processed dairy and industrial convenience meals, which occupy the top tiers of the FOMIC-Py. It should only be considered an exposure ranking tool that is required for future food safety exposure; it does not currently establish causal connections with specific illnesses.

### Methodological Justification and Model Scope

The current study combines national food consumption statistics with harmonized concentration data from the literature to estimate dietary exposure to MPs, employing an integrative modeling approach. Several decisions regarding methodology were made to guarantee transparency, comparability, and reproducibility in light of the current state of knowledge. Since the available research papers exhibit severe methodological heterogeneity in terms of analytical techniques (μFTIR; μRaman; Pyrolysis–gas chromatography–mass spectrometry, Py-GC/MS; Laser Direct Infrared, LDIR) [120,121,122], particle size detection limits, sample preparation processes, and reporting units (MPs/kg, MPs/L, MPs/item), a weighted meta-analysis strategy was not employed. These disparities render it impossible to apply conventional meta-analytic weighting techniques using a structured process-packaging proxy methodology. Consequently, the operational representative value for each food category was selected as the midpoint between the lowest and maximum reported concentration values. Instead of trying to estimate an accurate population mean, this technique offers a reproducible and assumption-minimizing metric that maintains the relative ordering of food categories based on their MP burden without relying excessively on a single extreme value.

LDA was applied on aggregated consumption-weighted category data instead of individual concentration measurements, despite the fact that original MP concentration data are not normally distributed. Instead of establishing a prediction model, the exploratory and classificatory aim of LDA in this study was to identify multivariate dietary trends that differentiated Northern and Southern Italy. When applied to standardized variables and large aggregated datasets, LDA is known to be fairly adaptable to moderate deviations from normality. Nevertheless, in order to further validate regional differences, future studies might explore alternative multivariate techniques such as machine learning classifiers, PERMANOVA, or partial least squares discriminant analysis (PLS-DA).

The current model calculates potential dietary exposure as the yearly quantity of MP particles consumed by each family. Internal dose, bioavailability, toxicokinetics, and health risk are not estimated. Instead of implying causation, references to disease associations are given to provide biological plausibility. Accordingly, this study should be interpreted as an exposure assessment framework rather than a formal human health risk.

The use of modeled estimations for categories with lack of information, real-world variability in food contamination, analytical variability among studies, and consumption data estimation represent a few of the sources of uncertainty. Exposure estimates are probably conservative as a result of this accumulated uncertainty. The identification of major factors contributing to overall consumption and the order of importance of food categories, nevertheless, are resilient to these uncertainties. As a result, the method is more appropriate for prioritization and comparability than for precise estimation of the real individual exposure. We explicitly assert that the FOMIC-Py framework is not a completely confirmed prediction model of human internal dosage, but rather an experimental, hypothesis-generating tool. The absolute particle counts presented herein indicate estimated orders of magnitude due to the significant analytical variability in MP extraction and quantification techniques across the literature. Rather than providing accurate exposure metrics, this model’s main utility is in establishing a uniform, mechanism-based hierarchy to direct future targeted sampling and regulatory prioritization [44]. Health risk assessment and exposure assessment are two different scientific processes. Internal dosage, bioavailability, toxicokinetics, toxicodynamics, and dose-response relationships were not included, considering the reality that the present study predicts potential food consumption of MPs. As a result, the current data set cannot be utilized to reach any conclusions on adverse health effects [123,124].

## 5. Putative Limitations

Despite providing a methodical depiction of dietary exposure using the FOMIC-Py, the current study has a number of limitations that are inherent in the status of MP research. First, there is uncertainty in absolute particle counts due to the variety of analytical techniques used in the reviewed literature (i.e., different detection limits between μFTIR and μRaman spectroscopy) [117,118,119]. We have used a sensitivity analysis and a Quality of Evidence exposure system to address this. Second, the FOMIC-Py does not evaluate biological uptake directly, yet it does render it easier to identify high-exposure food patterns. In this section the connections between the “Microplastic Syndrome” and the “Chicago Cluster” [6] that were previously suggested in our research are addressed as theoretical frameworks for risk characterization.

Process-proxy interpolations are the basis of the MM used for categories without empirical data, such as convenience soups and some processed meat. These are essential for giving an exhaustive overview of the Italian diet, but they are theoretical approximations requiring particular empirical validation. The European Food Safety Authority (EFSA) Comprehensive European Food Consumption Database’s standardized annual food intake data and Food and Agriculture Organization of the United Nations (FAO) dietary statistics were combined with concentration ranges provided in the literature to provide estimates of MP exposure. In order to tackle this, we performed a sensitivity study (Table S5), which verifies that the hierarchical structure of food contamination stays constant even when absolute concentrations may change.

It is not appropriate to consider the PLS-DA classification performance measurements as confirmation of predictive accuracy in actual populations. Instead, they demonstrate the harmonized exposure dataset’s internal coherence [24,25,26].

Lastly, the “Microplastic Syndrome” and the “Chicago Cluster” are provided here as well-established conceptual theories concerning the clinical consequences [6]. Despite exposure patterns being described, there is no direct toxicological or epidemiological proof of causality in this study. Future studies must use thorough biomonitoring to reduce the gap between the high-exposure levels in our pyramid and long-term health effects.

MP intake is not a one-off event but a systemic part of current diets. Combining EFSA-based consumption patterns with experimentally validated concentration levels, exposure varies significantly between regions due to different eating habits that intensify particular contamination pathways. The majority of annual intake is provided for by a small number of high-consumption, contamination-prone foods, mainly dairy, drinks, and cereals; nevertheless, significant proof gaps in other high-intake categories indicate that current estimates are conservative. These findings demonstrate that the structure of consumption patterns determines regional exposure more than specific meals, and they establish definitive goals for risk exposure, mitigation techniques, and specialized measurement. Holistic strategies must address both food composition and the specific sources of contamination Representing the most effective approach to decrease human MP intake is by focusing control and monitoring operations on high-impact categories.

We recognize that an underestimating of NP exposure may result from the lack of a uniform lower size limit in the literature (varying from 1 µm to 100 µm, as illustrated in Table S2). Therefore, rather than reflecting an absolute total concentration, our model represents a baseline of distinguishable micro-scale particles. FOMIC-Py is probably a conservative baseline, even if it provides a strong hierarchy based on micro-scale particles (>1 µm). NP concentrations may be orders of magnitude greater, according to emerging data, especially in highly processed liquid matrices including milk and mineral water.

Third, concepts such as the “Chicago Cluster” and the “Microplastic Syndrome” are only presented here as theoretical, upstream epidemiological hypotheses regarding potential biological plausibility. The estimated exposure values are insufficient to determine real clinical results, considering this study provides no direct toxicological, mechanistic, or clinical evidence of causation [123,124].

## 6. Future Trends and Regulatory Implications

The FOMIC-Py reflects an important transition from tracking ambient “background” contamination to highlighting industrial processing as a potential contributor to human exposure. Future trends point to a shift from conventional polymers toward bio-based alternatives as global food systems lean increasingly toward ultra-processing. Nevertheless, since Level 5 products, such as bottled beverages and heat-sealed ready meals, exhibit a high MP density, the industry must prioritize the mechanical and thermal stability of these materials to avoid a “substitution paradox” in which bio-fragments simply replace synthetic ones. Furthermore, with the aim to address the “analytical gap” concerning NPs, which presently constitute the invisible base of the FOMIC-Py, optical microscopy must provide a path to high-throughput methods like LDIR imaging and Pyrolysis-GC/MS. The current EU regulations on Food Contact Materials (FCM) lack specific migration limits for MPs, so regulatory evolution must consider this transition [2,125,126]. Our findings of a 9% higher exposure risk in Northern Italy—caused by particular industrial and dietary patterns—highlight the importance of region-specific safety standards and “Plastic-Neutral” certifications for plants using advanced filtration. Ultimately, incorporating the FOMIC-Py for “MP-exposure scoring” in public health campaigns could enable consumers to drive a market-led reduction in over-processed products. To convert this diagnostic framework into a predictive model for global food safety, food technology, polymer science, and toxicology must be holistically integrated [125].

## 7. Conclusions

The current approach should not be regarded as a risk assessment for human health considering that it only evaluates a potential dietary exposure [123,124].

With classification accuracy reaching up to 100% in specific categories, several of the food categories demonstrated considerable discriminant ability. The integrated multivariate consumption profiles unique to each food category, rather than specific foods, were responsible for geographic variations. Since they provide a theoretical, upstream hypothesis about chemical safety, these geographical exposure patterns should not be taken as conclusive clinical proof. Although conceptual frameworks such as the “Chicago Cluster” or “Microplastic Syndrome” have been put forth in narrative reviews to map possible cardiometabolic and inflammatory risks, this study offers no direct toxicological, epidemiological, or biomonitoring data to support a causal relationship between human pathogenesis and dietary MP ingestion. Rather than a confirmed clinical risk differential, the found 9% greater estimated exposure in Northern Italy is due to differences in commercial food consumption statistics. By rendering it possible to scientifically describe regional dietary habits, this approach may help with nutritional monitoring and regional food policy. Having been considered, the findings confirm the significance of discriminant methods in interpreting high-dimensional food consumption data into significant spatial patterns.

The FOMIC-Py does not constitute a quantitative risk-assessment model but instead an experimental framework for prioritization. Food categories that could benefit from more analytical studies and coordinated monitoring are identified by the proposed hierarchy. The current study is not able to determine a causal relationship, quantify internal exposure, or determine the risk to human health, despite the observed patterns being consistent with a possible contribution of industrial processing and packaging to the incidence of dietary MPs. To confirm the proposed methodology and establish its suitability for regulatory decision-making, additional studies based on standardized analytical procedures, biomonitoring data, and prospective exposure assessment would be essential.

## Figures and Tables

**Table 1:** Categorization criteria for the Food Microplastics Pyramid (FOMIC-Py): Defining levels based on industrial processing intensity and packaging interaction.

Level	Classification	Industrial Processing Intensity and Packaging Interaction	Key Food Groups
1	Raw/untreated	Negligible; direct harvest, minimal or no plastic packaging; short supply chains	Tap water (filtered), home-grown produce
2	Low processing	Primary washing and sorting, limited cold-chain packaging, seasonal local distribution	Fresh vegetables, fruits, fresh meats (unprocessed)
3	Moderate processing	Moderate; bulk handling and distribution, simple mechanical washing, standard industrial packaging (PE, PP, films, coats)	Grains, flour, sea salt, legumes
4	High processing	High; secondary packaging, industrial grinding of filtration, prolonged storage in plastic-lined containers (i.e., PET, Tetra Pak)	Dairy products, processed fruit juices, refined oils
5	Ultra-processed	Extreme; multiple contact points with polymer surfaces, high-speed mechanical bottling, thermal sealing of plastic membranes, extensive additives	Bottle water, soft drinks, canned meats

In order to minimize data bias, table salt was methodically excluded from the analysis of the 56 food categories that were initially monitored within the household matrix, providing 55 operational factors for the final discriminant models.

**Table 2:** Estimated dietary exposure to MPs per serving across different food classes *.

Food Category	MPs (kg^−1^ or L^−1^)	Serving Size (g or mL)	MPs/Serving	Quality of Evidence ^a^	References
Crustaceans	5500	100	550.0	Medium	[33,34,35,36]
Potatoes	1550	200	310.0	Low	[37,38]
Yogurt	2200	120	264.0	Medium	[38,39,40]
Canned meat	3000	80	240.0	Low	MM
Pre-packaged soup	1450	150	217.5	Low	MM
Mineral water	800	250	200.0	High	[41,42,43,44,45,46]
Dry vegetables	2000	100	200.0	Low	[47,48]
Powdered milk	5500	30	165.0	Low	[27,49]
Non-alcoholic drinks (Nad)	750	200	150.0	High	[50,51,52,53,54,55,56]
Game	1150	100	115.0	Low	MM
Pastries	1100	100	110.0	Low	[50]
Eggs	2050	50	102.5	Medium	[38,54,57,58]
Fresh/Frozen legumes	1350	75	101.3	Low	[59,60]
Bananas	800	120	96.0	Low	[38,41]
Cheese	1257	75	94.3	Medium	[38,41,61]
Ice cream	1750	50	87.5	Low	[62,63]
Canned Tomatoes	550	120	66.0	Low	[38,41]
Pasta	650	100	65.0	Low	[38,41]
Beer	275	200	55.0	Medium	[38,42,52,64]
Fruit Juice	275	200	55.0	Medium	[51,65,66]
Rice	550	100	55.0	Medium	[38,67,68]
Frozen fruit	550	100	55.0	Low	MM
Bread and breadsticks	950	50	47.5	Medium	[38,41,69]
Milk	360	125	45.0	High	[38,52,61,70,71]
Fresh/Frozen fish	275	150	41.3	Medium	[33,38,72]
Biscuits	1250	30	37.5	Low	[73]
Fresh Vegetables	175	200	35.0	Low	[47,48]
Tea	550	50	27.5	High	[38,64,74,75,76]
Processed meat	550	50	27.5	Medium	[14,77,78]
Jams	900	30	27.0	Low	MM
Grapes and Strawberries	175	150	26.3	Low	[79,80]
Fresh Tomatoes	175	150	26.3	Medium	[81,82,83]
Poultry	175	100	17.5	High	[84,85,86,87,88]
Fruit in syrup	500	30	15.0	Low	[89]
Wine	105	120	12.6	Low	[38,42]
Calf	110	100	11.0	Low	[78]
Butter	1050	10	10.5	Medium	[39,70,90]
Pork	105	100	10.5	Low	[14,38]
Flour	170	60	10.2	Low	[91,92]
Seed oil	850	10	8.5	Medium	[41,93,94,95]
Coffee	275	30	8.3	Medium	[38,66,96]
Apples	55	150	8.3	Medium	[38,47,48]
Pears	55	150	8.3	Low	[47,48]
Olive oil	550	10	5.5	Medium	[41,93,94,97]
Salt	5500	1	5.5	Medium	[43,67,98,99]
Beef	55	100	5.5	Low	[14,38]
Canned fish	1100	80	88.0	Medium	[100,101,102,103]
Dry Fruit	150	30	4.5	Low	[104]
Citrus fruit	275	150	4.1	Low	[47]
Sugar	720	5	3.6	Medium	[67,105,106,107]
Margarine	300	10	3.0	Low	MM
Dry legumes	55	50	2.8	Low	MM
Liquors	55	30	1.7	Low	[56]
Horse meat	13	100	1.3	Low	MM
Lamb meat	10	100	1.0	Low	[108,109]
Lard	6	10	0.1	Low	MM

* MM derived from serving size assumptions and reported concentration data rather than direct experimental quantification. Based on process-proxy interpolation, MM are cautious estimates (refer to Equation (S1) in Supplementary Materials). In accordance with the cautious principle in exposure assessment, this approach was only used for categories with high dietary relevance but no empirical MP proof. ^a^ Quality of Evidence is classified as follows: Low/MM (measures modeled by analogy), Medium (limited or outdated studies), and High (several independent studies).

**Table 3:** Estimated total MP concentrations per serving across various food categories and their contribution to dietary Intake ^a^.

Food Category	MPs per Serving	MP Concentration Level ^b^
Crustaceans	550	Very High
Potatoes	310	Very High
Yogurt	264	Very High
Canned meat	240	Very High
Pre-packaged soup	218	Very High
Dry vegetables	200	Very High
Mineral Water	200	Very High
Powdered milk	165	Very High
Non-alcoholic drinks (Nad)	150	Very High
Game	115	Very High
Pastries	110	Very High
Eggs	103	Very High
Fresh/Frozen legumes	101	Very High
Bananas	96	High
Cheese	94	High
Canned fish	88	High
Ice cream	88	High
Canned tomatoes	66	High
Pasta	65	High
Rice	55	High
Frozen fruit	55	High
Fruit juice	55	High
Beer	55	High
Bread and breadsticks	48	Moderate
Milk	45	Moderate
Fresh/frozen fish	41	Moderate
Biscuits	38	Moderate
Fresh vegetables	35	Moderate
Processed meat	28	Moderate
Tea	28	Moderate
Jams	27	Moderate
Grape and Strawberries	26	Moderate
Fresh tomatoes	26	Moderate
Poultry	18	Low
Fruit in syrup	15	Low
Wine	13	Low
Calf	11	Low
Pork	11	Low
Butter	11	Low
Flour	10	Very low
Seed oil	9	Very low
Coffee	8	Very low
Apples	8	Very low
Pears	8	Very low
Beef	6	Very low
Olive oil	6	Very low
Salt	5	Very low
Dry fruit	5	Very low
Citrus fruit	4	Very low
Sugar	4	Very low
Margarine	3	Very low
Dry legumes	3	Very low
Liquors	2	Very low
Horse meat	1	Very low
Lamb meat	1	Very low
Lard	0.1	Very low
Total Cumulative Intake	3941 MPs/intake	

^a^ According to SINU, serving sizes were based on reported intake levels for the Italian population. MP concentrations were expressed as MPs/kg for solid foods and MPs/L for liquids. ^b^ Very High: ≥100 MPs/serving; High: 50–100 MPs/serving; Moderate: 20–50 MPs/serving; Low: 10–20 MPs/serving; Very low: ≤10 MPs/serving.

**Table 4:** Differences in pooled food categories between Northern and Southern Italy based on canonical discriminant analysis ^a^ (explained variance = 94.7%).

Food Category	Number of Variables	Wilks’ λ	*p*-Value	Canonical Correlation (Rc)
Meat and animal derivatives	9	0.21	0.012	0.89
Sea foods	4	0.34	0.003	0.81
Cereals and derivatives	6	0.47	0.107	0.73
Sugar, pastries, desserts	4	0.44	0.015	0.75
Fats and servings	5	0.52	0.064	0.88
Dairy	5	0.23	0.001	0.89
Fruit	8	0.21	0.013	0.89
Vegetables and legumes	7	0.10	0.001	0.95
Beverages	5	0.31	0.008	0.90
Total ^a^	55	0.021	<0.001	0.99

^a^ Salt was not included in the analysis. Variables are expressed as annual household MP intake (MPs/year). Values correspond to standardized discriminant coefficients and pooled within-group correlations (structure matrix).

**Table 5:** Main polymeric composition of MPs detected in selected food categories *.

Food Category	Predominant Polymers Identified
Cheese	PA, PE, PET, PP
Fish	PA, PE, PP, PVC, PS
Milk and yogurt	PA, PE, PET, PP
Non-alcoholic drinks (Nad)	PE, PET, PP
Mineral water	PE, PET, PP
Fruit	PE, PET, PP, PS, PVC
Vegetables	PE, PET, PP, PS, PVC

* The food categories reported in this table represent selected and representative classes of the analyzed food items.

**Table 6:** Annual MP intake per family across food categories in Northern and Southern Italy (Mean ± SD) ^a^.

Food Category	Northern Italy	Southern Italy
	MPs/Year	MPs/Year
Bread and breadsticks	115,259 ± 11,437	100,199 ± 12,648
Yogurt	99,495 ± 10,143	73,060 ± 8741
Pasta	79,324 ± 9645	87,242 ± 8802
Milk	74,862 ± 5264	75,780 ± 12,675
Mineral water	67,110 ± 16,024	66,036 ± 10,017
Potatoes	57,118 ± 8706	61,338 ± 10,997
Biscuits	49,890 ± 4187	46,331 ± 5347
Eggs	42,409 ± 6556	46,349 ± 6526
Cheese	37,914 ± 3451	31,322 ± 3804
Sugar	36,972 ± 1527	47,245 ± 8354
Ice cream	23,144 ± 2375	18,455 ± 3888
Olive oil	18,961 ± 3764	18,265 ± 2522
Dry vegetables	18,100 ± 3065	15,636 ± 2630
Jams	16,853 ± 3611	13,336 ± 2081
Processed meat	16,033 ± 1377	12,660 ± 2605
Fresh legumes	15,981 ± 1186	18,679 ± 4260
Bananas	15,930 ± 1419	15,818 ± 2339
Canned tomatoes	13,977 ± 2531	17,235 ± 5215
Rice	12,313 ± 2388	11,875 ± 1657
Beer	11,605 ± 894	11,270 ± 2763
Flour	8152 ± 1655	9804 ± 1468
Seed oil	9180 ± 1445	8879 ± 2541
Fresh vegetable	8796 ± 815	6582 ± 1468
Non-alcoholic drinks (Nad)	8783 ± 2153	7354 ± 1441
Fresh/frozen fish	8666 ± 1448	10,950 ± 1165
Pastries	8553 ± 1120	7490 ± 1066
Poultry	8229 ± 1844	8954 ± 1609
Game	8036 ± 1752	6879 ± 1624
Powdered milk	6256 ± 1468	4650 ± 1355
Butter	6129 ± 1668	4410 ± 738
Fresh tomatoes	6092 ± 1059	7325 ± 1171
Fruit in syrup	5794 ± 878	4932 ± 852
Wine	5530 ± 408	4081 ± 715
Pre-packaged soup	5148 ± 920	3849 ± 2081
Canned meat	4613 ± 833	4227 ± 1479
Coffee	3572 ± 549	3578 ± 425
Fruit juice	3218 ± 486	3013 ± 558
Grapes and strawberries	3025 ± 494	2515 ± 381
Canned fish	2681 ± 428	1880 ± 707
Tea	2668 ± 265	2585 ± 482
Apples	2003 ± 301	1947 ± 216
Calf	1988 ± 454	2330 ± 232
Crustaceans	1788 ± 641	3350 ± 1122
Pork	1667 ± 392	1895 ± 306
Citrus fruit	962 ± 133	787 ± 110
Pears	796 ± 180	1036 ± 240
Beef	745 ± 149	498 ± 165
Frozen Fruit	660 ± 235	890 ± 169
Dry fruit	463 ± 71	322 ± 120
Margarine	405 ± 131	499 ± 178
Dry legumes	219 ± 30	276 ± 96
Liquors	188 ± 59	137 ± 38.6
Lamb	10 ± 3	27 ± 12.8
Horse meat	10 ± 6	14.0 ± 18.7
Lard	3 ± 1	2.0 ± 0.6

^a^ Salt was excluded from the analysis, as its consumption exceeds direct dietary intake. Values represent estimated annual household intake derived from harmonized concentration data and national consumption statistics. Standard deviations reflect propagated variability from concentration ranges and consumption data.

## Data Availability

No new data were created or analyzed in this study. Data sharing is not applicable to this article.

## Supplementary Materials

The following supporting information can be downloaded at: https://www.mdpi.com/article/10.3390/toxics14070578/s1, Figure S1: PRISMA flow diagram, detailing the number of records identified, screened, included and excluded at each stage of the review; Table S1: Reference empirical range (cluster foods); Table S2: Household MP Intake: Mean, minimum, and maximum values across food categories; Table S3: Classification and numerical distribution of Italian food items according to commodity group and product sub-type; Table S4: Summary of canonical discriminant analysis for major food categories across Italian regions; Table S5: Statistical comparison between minimum and maximum exposure scenarios across food categories; Table S6. Metrics for Model Performance (Stratified 10-Fold CV); Table S7. Cross-Validated Confusion Matrix (Frequencies and Percentages); Table S8. Classification Report by Region; Table S9. Variable Importance Measures (Top 15 VIM).

## Abbreviations

The following abbreviations are used in this manuscript:

CAPI	Computer-Assisted Personal Interviewing
EFSA	European Food Safety Authority
FAO	Food and Agriculture Organization of the United Nations
FCM	Food Contact Materials
FOMIC-Py	Food Microplastic Pyramid
FTIR	Fourier-Transform Infrared Spectroscopy
HDPE	High-Density Polyethylene
ISTAT	Italian National Institute of Statistics
LDA	Linear Discriminant Analysis
LDIR	Laser Direct Infrared Spectroscopy
LOD	Lower Limit of Detection
MDI	Mean Decrease in Gini
MM	Modeled Measure
MPs	Microplastics
Mt	Million Tonnes
Nad	Non-Alcoholic Drinks
NPs	Nanoplastics
OOB	Out-of-Bag
PA	Polyamide
PE	Polyethylene
PET	Polyethylene Terephthalate
PLS-DA	Partial Least Squares Discriminant Analysis
PP	Polypropylene
PS	Polystyrene
PVC	Polyvinyl Chloride
Py-GC/MS	Pyrolosis-Gas Chromatography-Mass Spectrometry
Rc	Canonical Correlation
RF	Random Forest
SINU	Italian Society of Human Nutrition
VIM	Variable Importance Measures
VIP	Variable Importance in Projection
μFTIR	Micro-FTIR Spectroscopy
μRaman	Micro-Raman Spectroscopy
