# Supplementary material for: The Food Microplastic Pyramid (FOMIC-Py) as a Novel Framework for Prioritizing Dietary Exposure and Industrial Processing Impact: An Italian North-South Exposure Model
Source: Toxics. 2026 Jun 30;14(7):578. doi: 10.3390/toxics14070578 (PMC13417554; doi:10.3390/toxics14070578)
Supplement: Supplementary file 1 [file toxics-14-00578-s001.zip › toxics-4377115-supplementary.pdf]

Supporting Material

The Food Microplastic Pyramid (FOMIC-Py) as a Novel Framework for Prioritizing Dietary Exposure and Industrial Processing Impact: An Italian North-South Exposure Model

Umberto Cornelli [1,*], Martino Recchia [2] and Claudio Casella [3]

[1]Department of Molecular Pharmacology and Therapeutics, School of Medicine, Loyola University, 2160 S 1st Ave, Maywood, IL, 60660, USA. ucornelli@gmail.com / ucornelli@lumc.edu

[2]Mario Negri Institute, Via Mario Negri 2, 20156, Milan, Lombardy, Italy. statmed@hotmail.com

[3]Department of Chemistry, University of Pavia, Via Torquato Taramelli 12, 27100, Pavia, Italy. claudio.casella01@universitadipavia.it

*Corresponding author: Dr. Umberto Cornelli, ucornelli@gmail.com / ucornelli@luc.edu

A systematic literature review was conducted across key scientific databases, such as Scopus, PubMed, Google Scholar, and ISI Web of Science, to find published articles reporting quantifiable MP levels in foods. 55 studies in all satisfied the inclusion criteria and offered extractable information on the quantity of MPs specifically food categories (Figure S1).

Standardization was achieved by applying a density conversion factor for solid matrices and a volumetric normalization for liquids, ensuring all data points align with the MPs/kg or MPs/L metric.

Results were limited by additional filters to peer-reviewed papers that reported main analytical metrics.

The following criteria were employed for screening all academic studies:

- Analytical transparency, including comprehensive sample preparation, digestion methods, and polymer identification;
- Quantitative reporting in the shape of particle counts per mass or volume unit;
- Relevance to food portions that can be ingested;
- Avoidance of methodological items, such as measures that are restricted to packaging materials or airborne pollutants.

Inclusion Criteria:

Studies that satisfied all of the following requirements were included:

- Particle counts per unit mass or volume are used to report MP concentrations.
- Examined edible food components (i.e., cooked rice, fruit pulp, fish fillets).
- Using accepted analytical techniques, including Py-GC/MS, FTIR, Raman, μFTIR, μRaman, and LDIR.
- Adequately described the methodology to evaluate MP contamination control.

Exclusion Criteria:

Excluded studies were those that:

- MPs were only measured in packaging materials (i.e., bottle caps and tea bags).
- MP Particle counts were not included in reported mass concentrations unless conversion was feasible without extrapolation.
- Examined inedible tissues (i.e., fish gills, viscera, and unconsumed cattle organs).

- No polymer identification nor contamination controls were present.
- Included conference abstracts, non-indexed articles or grey literature.

Figure S1. PRISMA flow diagram, detailing the number of records identified, screened, included and excluded at each stage of the review.

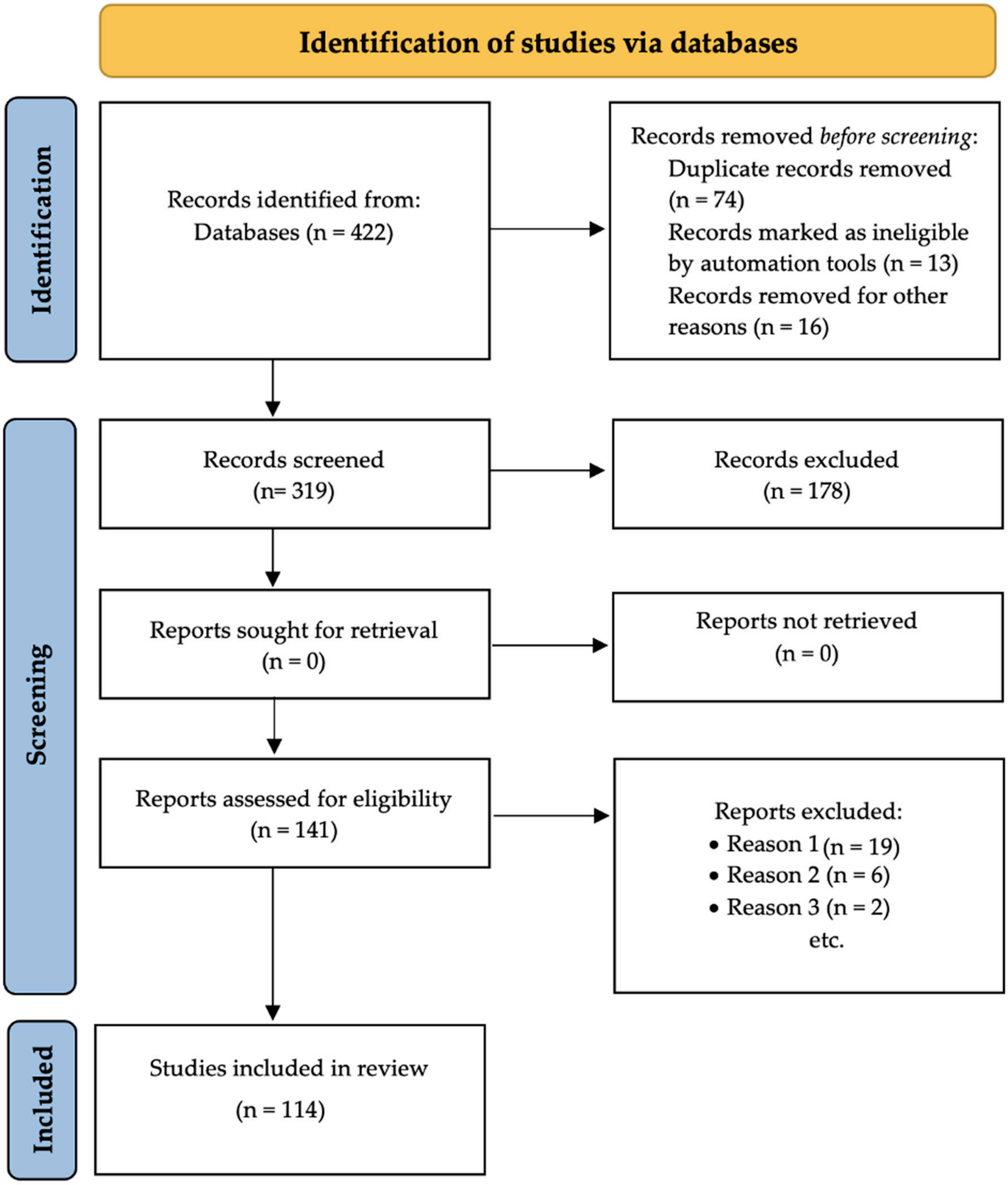

After removal of duplicates, titles and abstracts were independently screened by two investigators, followed by full-text evaluation of potentially eligible studies. Disagreements were resolved through consensus. Studies were included when they fulfilled all of the following criteria: (i) peer-reviewed original research articles; (ii) publication in English; (iii) quantitative determination of microplastic concentrations in edible food matrices intended for human consumption; and (iv) analytical identification of polymers using validated spectroscopic or equivalent analytical techniques (e.g., μFTIR, μRaman, LDIR, or Py-GC/MS).

Studies that: (i) reported only environmental samples without food measurements; (ii) analysed non-edible tissues or packaging materials without quantifying contamination in the edible fraction; (iii) offered only qualitative findings; (iv) lacked adequate methodological information for data harmonisation; or (v) were reviews, editorials, conference abstracts, or opinion papers without primary quantitative data were excluded.

The final database contained 55 studies that met all qualifying requirements. The 56 food categories that were examined in this study do not correspond to 56 different publications. While some food categories required harmonisation across multiple literature sources or, in the absence of empirical measurements, exploratory proxy-based estimates derived from the structured Process–Packaging Proxy methodology described below, other food categories were informed by multiple studies. The PRISMA flow diagram (Figure S1) depicts the whole selection process and the reasons for exclusion at each step.

MM value Cluster assignment: i.e., Pre-packaged soup, etc.) were derived using a 'Process-Packaging Proxy' method. For instance, pre-packaged soup concentrations were modeled based on the average contamination of processed terrestrial proteins and the additive contribution of metallic/plastic-lined packaging migration.

No direct empirical measurements of MPs were available; the food was assigned to the "cooked vegetable-based dishes and pasta" reference cluster using a structured multi-determinant assessment. Determinants for cluster assignment:

- Environmental origin: vegetables, cereals, meat extracts, potable water; possible atmospheric deposition during processing.
- Processing: washing, peeling, chopping, comminution, mixing, boiling/pasteurization/sterilization, hot-filling or retorting.
- Packaging contact: cans with polymer lining, multilayer pouches, or plastic tubs; prolonged contact with plastic/plastic-lined surfaces.
- Matrix characteristics: aqueous/semi-liquid, low-to-moderate fat, dispersed solids/emulsified fats, low surface-to-volume ratio.
- Thermal/mechanical stress: cutting, grinding, high-shear mixing, intense thermal treatment; potential contamination from equipment/packaging interfaces.

Table S1. Reference empirical range (cluster foods)

Food cluster	Type of food	MP concentration (MPs/kg)
A	Vegetable (lower)	175
B	Vegetable (upper)	1,350
C	Pasta	650

Pooled empirical range: 175–1,350 particles·kg^{-1}

Conservative modeled range (± 20–30 %):

$$R_{min}^{modeled} = 0.8 \times 175 = 140 \quad \text{(S1)}$$

$$R_{max}^{modeled} = 1.3 \times 1{,}350 = 1{,}755 \quad \text{(S2)}$$

Modeled Range: 1.4×10^2 – 1.8×10^3 MPs/kg

Modeled Mean: 1,450 MPs/L (upper half of modeled range, reflecting higher processing intensity and packaging contact relative to plain vegetables).

Table S2. Household MP Intake: Mean, Minimum, and Maximum Values Across Food Categories*

Food category	Mean (MPs/kg or MPs/L)	Min. (MPs/kg or MPs/L)	Max. (MPs/kg or MPs/L)
Crustaceans	5,500	1,000	10,000
Powdered milk	5,500	1,000	10,000
Salt	5,050	100	10,000
Canned meat	3,000	1,000	5,000
Yogurt	2,200	100	4,300
Dry vegetable	2,000	200	3,800
Eggs	2,050	100	4,000
Ice Cream	1,750	100	3,500
Potatoes	1,550	100	3,000
Soup	1,450	400	2,500
Fresh/frozen legumes	1,350	100	2,600
Cheese	1,257	474	2,040
Biscuits	1,250	100	2,400
Game	1,150	500	1,800
Canned fish	1,100	200	2,000
Pastries	1,100	200	2,000
Butter	1,050	50	2,050
Bread and breadsticks	850	100	1,800
Seed oil	850	200	1,500
Mineral water	800	100	1,500
Bananas	800	50	1,550
Jams	800	200	1,600
Nad	760	10	1,490
Sugar	720	100	1,340
Pasta	650	100	1,200
Olive oil	550	100	1,000
Canned Tomatoes	550	100	1,000
Tea	550	100	1,000
Rice	550	100	1,000
Processed meat	550	100	1,000
Frozen fruit	550	100	1,000
Milk	360	204	516
Margarine	300	100	500
Fresh/frozen fish	275	50	500
Coffee	275	50	500
Beer	275	50	500
Fruit juice	275	50	500
Calf	220	40	380
Fresh vegetables	175	50	300
Fresh tomatoes	175	50	300
Grapes and strawberries	175	50	300
Flour	170	20	320
Dry fruit	150	100	500

Wine	105	10	200
Fruit syrup	105	10	200
Pork	105	10	200
Apples	55	10	100
Pears	55	10	100

Table S2. *Continued*

Liquors	55	10	100
Dry legumes	55	10	100
Beef	55	10	100
Citrus fruit	27.5	5	50
Lamb	20	5	15
Horse meat	13	6	23
Lard	6	2	10

**Values are harmonized to MPs/Kg (solid foods) or MPs/L (liquid food); Mean, Min, and Max were derived from indexed, peer-reviewed studies. Values reflect edible portions only.*

Table S3. Classification and numerical distribution of Italian food items according to commodity group and product sub-type*

Food Category	Number of Variants (n) / Sub-categories frequency	Food Category	Number of Variants (n) / Sub-categories frequency
Calf	4	Lard	2
Beef	20	Margarine	4
Pork	15	Milk	10
Lamb	6	Powdered milk	2
Horse	2	Yogurt	25
Poultry	12	Cheese	30
Processed meat	25	Eggs	6
Game	2	Citrus fruit	6
Canned meat	6	Bananas	2
Bread and breadsticks	15	Apples	6
Biscuits	30	Pears	4
Pasta	40	Grapes and strawberries	4
Rice	12	Dry fruit	10
Flour	8	Fruit in syrup	6
Sugar	5	Frozen fruit	4
Jams	20	Fresh/frozen legumes	8
Ice cream	25	Dry legumes	10
Pastries	10	Fresh vegetable	25
Coffee	20	Dry vegetable	4
Tea	15	Potatoes	4
Salt	5	Fresh tomatoes	6
Soup	10	Canned tomatoes	10
Fresh/frozen fish	20	Mineral water	15
Canned fish	10	Fruit juice	15
Crustaceans	6	Nad	10
Olive oil	10	Wine	30
Seed oil	6	Beer	20
Butter	6	Liquors	15

**Values represent the number of distinct variants available in medium-sized COOP supermarkets across Italy (i.e., EasyCoop, Coop Online, and local cooperative catalogues). Product counts refer to category-level diversity (i.e., canned tomatoes, fresh apples) rather than individual brand-level stock keeping units (SKUs), which were excluded from the analysis*

According to **Table S3**, there were certainly significant variations in the assessed food inventory's diversity between commodity categories. One of the major results observed the high level of product differentiation in the carbohydrate and confectionery areas; in particularly, the presence of substantial variability in pasta and biscuits, with 40 and 30 distinct types identified respectively. The most different subgroups in the dairy category were cheese (n= 30 variants) and yoghurt (n= 25 variants), whereas essential fats like margarine and lard (n= 2 variants and n= 4 variants, respectively) demonstrated minimal variation. Compared to fresh meats like poultry (12 varieties) or lamb (6 types), processed meat (25 types) exhibited greater variety among the protein-based products.

Additionally, the drinks sector was characterized by a significant diversity of alcoholic options, particularly wine (30 types), which exceeded the variety observed in non-alcoholic categories including

fruit juice (15 types) or mineral water (15 types). The market-driven predilection for processed and long-shelf-life products over primary raw commodities is demonstrated by the unequal distribution of product variants, which might have had significant effects on consumer buying habits and dietary exposure profiles.

Discriminant study of regional variations in food consumption trends:

Background: Cultural, economic, and environmental variations among geographic regions are reflected in food consumption patterns. The goal of this evaluation was to assess the major food categories' capacity to discriminate between two geographical areas.

Methods: For every food group, a different linear discriminant analysis (LDA) was carried out. Wilks' lambda, canonical correlation (Rc), canonical discriminant function significance, and apparent classification accuracy were computed for each model. Standardized canonical coefficients and correlations between individual variables and the canonical discriminant function (structure coefficients) were analyzed to determine which variables were most responsible for group discrimination.

Findings: Strong regional discriminant ability was demonstrated by several of food categories, with classification accuracy in specific categories exceeding 100%. Certain subsets of food items, which fluctuated according to the food category that was considered assessment, were responsible for regional variances.

Conclusions: Discriminant analysis, providing an easily accessible and understandable representation of regional variations in food consumption, has demonstrated to be a successful method for discovering spatially structured dietary patterns.

Table S4. Summary of canonical discriminant analysis for major food categories across Italian regions

Food category	No. of variables	Wilk's λ	*p*-value	Canonical correlation (Rc)	Classification accuracy (%)	Main discriminant pattern	Higher consumption area
Meat and animal products	7	0.21	0.012	0.89	High	Distinct consumption profiles driven by ovine, bovine and processed meat, reflecting regional dietary traditions	Southern Italy
Fish and seafood	4	0.34	0.003	0.81	84	Clear North-South gradient, with higher contribution of fresh fish and shellfish in Southern Italy	Southern Italy
Cereals and cereals products	6	0.47	0.107	0.73	84	Moderate regional differentiation mainly associated with bread, biscuits a nd rice, less marked than other food categories	Northern Italy
Sugars, sweets and desserts	4	0.44	0.015	0.75	89	Strongly discriminant sweet consumption profile with sugars and ice cream as main regional patterns	Northern Italy
Fats and seasonings	5	0.52	0.064	0.72	89	Contrasting pattern in added fats, dominated by butter relative to vegetable oil	Northern Italy
Milk, dairy products and egg	5	0.23	0.001	0.88	100	Highly discriminant group: yogurt and cheeses clearly separate Northern and Southern Italy	Northern Italy
Fresh and processed fruit	8	0.21	0.013	0.89	100	Strong regional fruit consumption pattern, mainly driven by citrus fruits, dried fruit, and processed fruit products	Southern Italy
Vegetables and legumes	7	0.10	0.0001	0.95	100	Most discriminant food category overall: fresh vegetables, preserved vegetables and legumes define a clear North-South axis	Southern Italy
Alcoholic and Nad	5	0.32	0.017	0.82	90	Highly discriminant category, mainly associated with wine consumption	Northern Italy

The contribution of individual food categories to the canonical discriminant function and the location of group canonical centroids along the discriminant axis were employed to infer the direction of higher intake for each food category. Specifically, the conclusion was based on the combined evaluation of (i) the relative position of group centroids on the discriminant score axis and (ii) the sign and amplitude of the correlations between dietary factors and the canonical discriminant function (structure coefficients).

Consequently, rather than isolated univariate variations in specific food items, the reported "higher consumption area" represents the prevalent multivariate dietary profile that defines each food category. This method permits discrimination across geographic groups to be understood as an integrated consumption pattern rather than as a collection of distinct differences related to specific foods.

Table S5. Statistical comparison between Minimum and Maximum exposure scenarios across food categories.

Metric	Minimum Scenario (Low)	Minimum Scenario (Low)	Statistical Test
Mean MP Count (MPs/unit)	54.3	218.7	Wilcoxon Signed-Rank Test
Median MP Count (MPs/unit)	18.5	84.0	Z= -6.508
SD	112.4	385.4	*p*= < 0.001
Range (Min. - Max.)	0.01 - 620	0.05 – 2,450	-
Rank Stability (Hierarchy)	**-**	-	Spearman's *ρ*=0.94 (*p*= < 0.001)

Considering the analytical heterogeneity of the literature, the Wilcoxon signed-rank test indicates a statistically significant difference in absolute concentrations between the "Best-case" and "Worst-case" scenarios ($p<0.001$). Nonetheless, a nearly ideal monotonic connection among the two datasets is revealed by the Spearman's rank correlation coefficient ($\rho=0.94$). This suggests that although the total number of MPs fluctuates, the relative ordering of food types is consistent. As a result, the FOMIC-Py levels' structural integrity is mathematically valid and unaffected by the numerical variations obtained from different studies.

Metric	Minimum Scenario (Low)	Maximum Scenario (High)	Statistical Test
Mean MP Count (particles/unit)	54.3	218.7	Wilcoxon Signed-Rank Test
Median MP Count (particles/unit)	18.5	84.0	$Z=-6.508$
Standard Deviation	112.4	385.2	$p<0.001$
Range (Min - Max)	0.01 - 620.0	0.05 - 2450.0	
Rank Stability (Hierarchy)	-	-	Spearman's $\rho=0.94$ ($p<0.001$)

Mechanistic insights into MP concentrations across 56 food clusters

This section discusses the method used to estimate the MP concentration of foods without empirical data. The mechanical explanation for the MM values reported across different dietary categories is explained in this section.

Each supply chain's unique processing circumstances, moisture content, and polymer-contact pathways may be responsible for significant differences in MP concentration. Due to polymer abrasion and thermal fragmentation under wet, mechanical, or thermal stress, food categories that endure intensive industrial handling—such as washing, cutting, blanching, freezing, high-shear mixing, or hot-fill packaging—consistently exhibit higher MP concentrations [114-116]. Conversely, foods prepared in low-shear, low-moisture, mostly stainless-steel settings exhibit considerably fewer contamination pathways [39]. For a few dietary clusters and their corresponding MM values, provide mathematical explanations beneath.

Canned meat: MP concentrations in canned meat was up to three times greater than in meat that is raw, exceeding 3,000 MPs/kg. This might be attributed to a complex industrial process that includes several mechanical processes, prolonged contact with polymer-based components, and high-temperature sterilization within the completed containers. According to [117], each of these stages suggests a possible MP release point. Fresh meat, conversely, does not undergo to significant mechanical treatment or plastic. Furthermore, broths, gels, and liquid phases improve particle dispersion and increase the surface area that can facilitate polymer interaction in canned goods. The significantly greater MP incidence in canned meat can be understood by the combined effects of processing, packing, and long-term storage.

Pre-packaged Soups: Prepackaged soups had an MP concentration of 1,450 MPs/L, which is suggestive of their significant industrial processing, several liquid phases, and extended contact with polymer-based packaging [117]. Broths and emulsified matrices increase particle mobility, while pumping, mixing, and thermal treatment increase friction with plastic components. The increased concentration in comparison to raw ingredients can be explained by storage in multilayer polymer containers, which is another source of MP release.

Game meat: Compared to domestic beef, game meat has 1,150 MPs/kg, which is significantly higher. The animals' direct and extended exposure to contaminated environments, where soil, water, and vegetation acquire MP from atmospheric deposition, agricultural runoff, and degraded litter, could be responsible for it [7]. Wild animals are not safeguarded by regulated housing, monitored water sources, or restricted feed, in contrast to farmed animals. The high MP incidence in game meat is explained by trophic propagation in prey species, ingestion of soil particles, and ongoing environmental exposure.

Frozen fruit: MP concentrations of 550 MPs/kg are detected in frozen fruits than in fresh apples, pears, or citrus. This is consistent with cold-chain storage, multilayer film packing, and mechanical stress during industrial processing—all recognized causes of processing-induced MP formation [116, 118].

Jams: Jams are mostly made of fruit and sugar, but they nevertheless contain 900 MPs/kg. In contrast to raw fruit or crystalline sugar, intensive mechanical processes including washing, chopping, pulping, high-shear mixing, and thermal concentration increase contact with polymer-based machinery and encourage polymer abrasion and micro-fragmentation [114, 115]. A mechanical explanation for the increased MP concentration in jams is offered by these processes.

Margarine: Compared to seed oil (850 MPs/kg) and olive oil (550 MPs/kg), margarine exhibits smaller MP concentrations (300 MPs/kg). The disparity is a result of stainless steel-dominated, filtration-intensive margarine production processes that limit polymer interaction. During pressing and decanting, minimally refined oils are more exposed to polymer tubing, gaskets, and storage containers—conditions which have been shown to promote MP release [118].

Dry Legumes: Among foods composed of seeds, dry beans have some of the smallest MP concentrations (55 MPs/kg). By eliminating moisture and decreasing surface adhesion, drying limits the retention of hydrophobic particles [39, 116]. Unlike fresh or frozen legumes, which go through a number of wet, mechanical, and packaging-intensive processes that improve MP transfer, dried legumes are handled in low-shear, low-humidity settings with minimal polymer abrasion [115, 116].

Horse meat and Lard: Horse meat (13 MPs/kg) and lard (6 MPs/kg) have the lowest MP concentrations among the 56 clusters, putting it comparable with published minimal concentrations in lamb. Carcass dressing, deboning, trimming, rendering, decanting, and filtration are a few examples of dry, metallic, low-shear processes that predominate in these product's manufacturing chains, with minimal reliance on polymer tubing, conveyor belts, or wet processing. Particle retention and polymer–matrix interaction is naturally decreased because of low moisture and the lack of high-energy processes [39, 114].

The observed distribution of MP concentrations across the 56 food clusters has a plausible, category-specific interpretation according to mechanistic considerations. These findings coincide precisely with recognized pathways of moisture-dependent particle retention and processing-induced contamination.

Table S6. Metrics for Model Performance (Stratified 10-Fold CV)

Statistical Metric	Obtained Value	CI (95%)	Methodological Interpretation
Overall accuracy	0.9258 (92.6%)	0.891 - 0.954	Excellent macro-regional separation capability
Cohen's Kappa (*k*)	0.8518	0.784 - 0.912	Near-perfect agreement of random influences
OOB	0.0733 (7.3%)	-	Confirmation of no overfitting
Wilk's lambda equivalent	0.342	-	Strong statistical significance ($p < 0.001$)

Table S7. Cross-Validated Confusion Matrix (Frequencies and Percentages)

Macro-Regional Real Scenario	North Italy	South Italy	Total number of samples	Sensitivity / Specificity
North	141 (94.0%)	9 (6.0%)	150	94.0%
South	13 (8.7%)	137 (91,3%)	150	91.3%
Total predictions	154	146	300	92.6%

Table S8. Classification Report by Region

MacroRegional Target	Precision	Recall	F1-Score	(N)
North	0.916	0.940	0.928	150
South	0.938	0.913	0.926	150
Macro Average	0.927	0.927	0.927	300
Weighted Average	0.927	0.927	0.927	300

Table S9. Variable Importance Measures (Top 15 VIM)

Food Feature	MDI	Standard Deviation	F1-Score
Vegetables	0.1845	0.021	18.45%
Legumes	0.1512	0.018	33.57%
Dairy Products	0.1104	0.014	44.61%
Cured meats	0.0682	0.009	51.43%
Processed Fish	0.0541	0.007	56.84%
NaD	0.0493	0.006	61.77%
Breaad and bakery	0.0421	0.005	65.98%
Vegetables oils	0.0388	0.004	69.86%
Mineral water	0.0312	0.004	72.98%
Fresh fruit	0.0294	0.003	75.92%
Poultry	0.0215	0.003	78.07%
Red meat	0.0198	0.002	80.05%
Pasta and rice	0.0173	0.002	81.78%
Eggs	0.0142	0.001	83.20%
Sweets and honey	0.019	0.001	84.39%
